# Quantitative and stability study of the evolution of a thermoelastic body

**DOI:** 10.1016/j.mex.2022.101983

**Published:** 2022-12-23

**Authors:** Pascal H. Zinsou, Guy Degla, Khalil Ezzinbi

**Affiliations:** a Institut De Mathematiques Et De Sciences Physiques (IMSP), 01 BP 613 PORTO-NOVO, Benin; b Universite cardi ayyad, facultedes sciences Semlalia, Departement de mathematiques, B.P.2390, Marrakesh, Morocco

**Keywords:** Integrodifferential equation, Maximal monotone operator, Semi group of contraction, Existence of global solutions, Spectral method stability, Semi group method

## Abstract

We prove the existence and uniqueness of a solution to a system of equations describing the evolution of a linear thermoelastic body by using a semi-group method. Moreover, the uniform exponential stability of the solution is shown in a particular case.

• With respect to the existence and uniqueness of the solution, we have defined a linear operator which generates a contraction semi-group and show that it is monotone maximal.
• With respect to the stability of the system, we have computed explicitly the expression of the solution of the system and show that the semi-group is uniformly exponentially stable in a particular case.

## Graphical abstract

**Figure:**
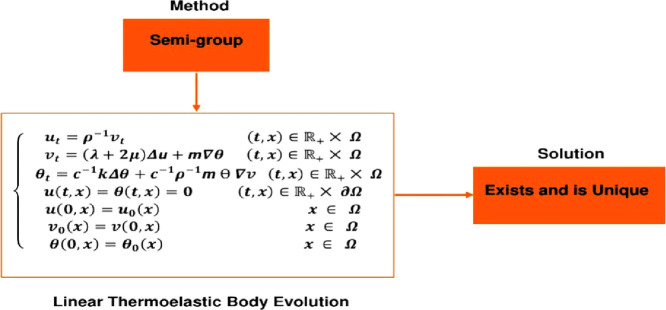

Specifications table

**Table:** 

Subject area:	Mathematics and Statistics
More specific subject area:	Analysis
Name of your method:	Semi group method
Name and reference of original method:	Technique of Bey et al. [E.J.D.E.78(2001)1–23] Nicaise method [Rendiconti Di Matematica, Ser-VII 23(2003)83–116]
Resource availability:	N.A.

**Method details**

## Introduction

In this paper, we are interested in the following system of equations describing the evolution of a linear thermoelastic body:

(1.1) $$\left\{ \begin{array}{cc} u_{t}=\rho^{-1}v_{t\;} & \left(t,x\right)\in R_{+}\times \Omega ,\\ v_{t}=\left(\lambda +2\mu \right)\Delta u+m\nabla \theta & \left(t,x\right)\in R_{+}\;\times \;\Omega ,\\ \;\theta_{t}\;=c^{-1}k\Delta \theta +c^{-1}\rho^{-1}m\;\theta \nabla v\; & \left(t,x\right)\in R_{+}\;\times \Omega ,\\ u\left(t,x\right)=\theta \left(t,x\right)=0 & \left(t,x\right)\in R_{+}\;\times \partial \Omega \\ u\left(0,x\right)=u_{0}\left(x\right) & x\;\in \;\Omega \\ v\left(0,x\right)=v_{0}\left(x\right) & x\;\in \;\;\Omega \\ \theta \left(0,x\right)=\theta_{0}\left(x\right) & x\;\in \;\;\Omega \end{array}\right.$$

where ρ>0 is the density of the body that ρ is constant, *θ* ˃ 0 the referential temperature, and c>0, k>0, m, λ, μ are constants, Ω is a nonempty open and bounded subset in $R^{n},$ u(t,x) the displacement, v(t,x) the momentum and θ(t,x) the temperature. Several mathematical models that come from physics (thermoelasticity motion) lead to the study of partial differential equations (PDEs) and sometimes evolution equations allowing mathematicians to describe the behavior of a quantity that depends on several variables. The equations of thermoelasticity describe the elastic and the thermal behavior of elastic, heat conductive media, in particular the reciprocal actions between elastic stresses and temperature differences. We consider the classical thermoelastic system where the elastic part is the usual second-order one in the space variable. The equations are a coupling of the equations of elasticity and of the heat equation and thus build a hyperbolic-parabolic system. Indeed, both hyperbolic and parabolic effects are encountered. Thermoelasticity is the elasticity of bodies resulting from an increase in temperature. Thermoelasticity is the appropriate model for the explanation of the decay of the amplitude of free vibrations of some elastic bodies. Since then a wide variety of results and applications have been obtained in many different fields. For instance, in [[3], [4], [5], [18]] synthetic tissues which mimic human bones are investigated, while in [16] cardiological tissues are considered. Besides a model of apples regarded as thermoelastic bodies is studied in [14,19].

The present investigation concerns thermoelastic bodies and their thermic behavior aiming to widen the range of applicable cases the theory can be applied to. In [1], Amar Herminna, Abdoulaye Sene and Serge Nicaise studied the existence, uniqueness and stability of a solution of this system with $v=\rho u_{t}$, λ+2μ=1, ck=1 and div[σ(u)]=Δu. In this work, we have shown the existence and uniqueness of the solution of this system in a general case and stability in a particular case by using a semi group method.

This work is presented in five parts. In the first part, we have presented the preliminaries, and the mathematical model of the system describing the evolution of a linear thermoelastic body in the second part. In the third part, we have shown the existence and uniqueness of the solution of the system through the methods of semi groups. In the fourth part, we have shown that the system is exponentially stable in a particular case, in fith we give an application and we have presented the section conclusion

**PRELIMINARIES**

Definition 2.1 **[2]**

Let X be a Hilbert space and Λ:D(Λ)⊆X→X be a linear operator. We say that Λ is monotone if 〈Λx,x〉≥0 for all x∈D(Λ). Λ is said to be maximal monotone if Λ is monotone and Im(I+Λ)=X.

The following theorem is variant of Lumer Philippe's theorem that is very useful to show the existence and the uniqueness of the solution of an evolution system.

Theorem 2.2 **[2]**

*Let*X*be a real Hilbert space and*Λ:D(Λ)⊆X→X*be a linear operator. If*−Λ*is maximal monotone on*X*, then*Λ*is the infinitesimal generator of a*$C_{0}$*-semi-group of contractions*${\left(T(t)\right)}_{t\geq 0}$*on*X.

Next we present some definitions and theorems of the stability of an evolution system.

Definition 2.3 **[13]**

We define the growth rate of a semi-group ${\left(T(t)\right)}_{t\geq 0}$ by

$$w_{0}\left(T\left(t\right)\right)=inf\left\{w\in R;\exists M\geq 1/∥T\left(t\right)∥\leq Me^{wt},t\geq 0\right\}.$$

Definition 2.4 **[13]**

We call spectral bound of an operator Λ, the number denoted by S(Λ) and defined by

s(Λ)=sup{Re(λ);λ∈σ(Λ)}.

Definition 2.5 **[[13], [17]]**

Let A be an infinitesimal generator of a $C_{0}$-semi group ${\left(T(t)\right)}_{t\geq 0}$. Then we have:

$$\underset{t>0}{\text{inf}}\frac{log∥T\left(t\right)∥}{t}=\underset{t\to +\infty }{\text{lim}}\frac{log∥T\left(t\right)∥}{t}\leq \frac{1}{t_{0}}log\left(r\left(T\left(t_{0}\right)\right)\right),$$

$$-\infty \leq s\left(A\right)\leq w_{0}\left(A\right)<+\infty ,$$

for each $t_{0}>0$ and $r(T(t_{0}))$ the spectral radius of $T(t_{0}).$

Definition 2.6 **[13]**

Let ${\left(T(t)\right)}_{t\geq 0}$ be a $C_{0}$-semi group on a Banach space X. ${\left(T(t)\right)}_{t\geq 0}$ is exponentially stable if and only if$\;w_{0}\left(T\right)<0$.

Proposition 2.7 **[15]**

*Let*${\left(T(t)\right)}_{t\geq 0}$*be a*$C_{0}$*-semi group on a Banach space X.*${\left(T(t)\right)}_{t\geq 0}$*is uniformly exponentially stable if and only if for some*p*,*1≤p<∞

$$\int_{0}^{\infty }∥T\left(t\right)\xi {∥}^{p}dt<\infty ,$$

for every ξ∈X.

## MATHEMATICAL MODEL

Let C be a homogeneous body having as referential configuration a nonempty, open and bounded subset Ω in R. The state of the body at the time t∈R, is characterized by two vector fields: the displacement u(t,x), the momentum v(t,x) and a scalar fields: the temperature θ(t,x). The system of equations describing the evolution of these three fields is:

(3.1) $$\left\{ \begin{array}{cc} u_{t}=\rho^{-1}v_{t\;} & \left(t,x\right)\in R_{+}\;\times \Omega \\ v_{t}=\left(\lambda +2\mu \right)\Delta u+m\nabla \theta & \left(t,x\right)\in R_{+}\times \Omega \\ \theta_{t}=c^{-1}k\Delta \theta +c^{-1}\rho^{-1}m\;\theta \nabla v & \left(t,x\right)\in R_{+}\;\times \Omega \\ u\left(t,x\right)=\theta \left(t,x\right)=0 & \left(t,x\right)\in R_{+}\;\times \partial \Omega \\ u\left(0,x\right)=u_{0}\left(x\right) & x\;\in \;\Omega \\ v\left(0,x\right)=v_{0}\left(x\right) & x\;\in \;\Omega \\ \theta \left(0,x\right)=\theta_{0}\left(x\right) & x\;\in \;\Omega \end{array}\right.$$

where ρ>0 is the density of the body specify that ρ is constant, θ>0 the referential temperature, and c>0, k>0, m, λ, μ are constants which characterized the thermoelastic properties of the body with *c*^2^=0 and $\nabla u\cdot \nabla (v_{t}-v)\leq 0$, ∇v=ρkθ with $\nabla u=\frac{\partial u}{\partial x}$ and $\Delta u=\frac{\partial^{2}u}{\partial x^{2}}$. Composite materials used as structural elements in high-tech fields (aerospace industry, automotive industry…) are subjected in many cases to thermal stresses (turbojet $1000^{c}$, supersonic combustion $1750^{c}$, missile cone…) The components of these composites do not expand in the same way. This difference in expansion coefficients can lead to plasticization or total failure. This justifies the need to determine the thermoelastic behavior. So, the present system is a generalization of the evolution of bodies in motion of thermoelasticity. We begin by rewriting the system (3.1) under the form of an abstract Cauchy problem in a suitably chosen Hilbert space. More precisely, let

$$H=\left[H_{0}^{1}\left(\Omega \right)\cap H^{2}\left(\Omega \right)\right]\times \left[L^{2}\left(\Omega \right)\right]\times L^{2}\left(\Omega \right),$$

be endowed with the mapping 〈.,.〉 defined by:

(3.2) $$\langle \left(u,v,\theta \right),\left(\tilde{u},\tilde{v},\tilde{\theta }\right)\rangle =\int_{\Omega }\left\{\rho^{-1}v\tilde{v}+\left(\lambda +2\mu \right)\nabla u.\nabla \tilde{u}+c^{-1}\theta \tilde{\theta }\right\}dx.$$

*Then the following proposition holds.*

Proposition 3.1 *The mapping 〈*.,.〉*defined on H by*(3.2)*is an inner product*.

Proof. We show that 〈.,.〉 is bilinear and symmetric.

Let $\left(u,v,\theta \right),\left(\tilde{u},\tilde{v},\tilde{\theta }\right),\left(u_{1},v_{1},\theta_{1}\right),\in H\times H\times H$ and α∈R.

Show first that 〈.,.〉 is symmetric.

$$\langle \alpha \left(u,v,\theta \right)+\left(u_{1},v_{1},\theta_{1}\right),\left(\tilde{u},\tilde{v},\tilde{\theta }\right)\rangle =\langle \left(\alpha u+u_{1},\alpha v+v_{1},\alpha \theta +\theta_{1}\right),\left(\tilde{u},\tilde{v},\tilde{\theta }\right)\rangle .$$

$$\;=\int_{\Omega }\left\{\rho^{-1}\left(\alpha v+v_{1}\right)\tilde{v}+\left(\lambda +2\mu \right)\left(\alpha \nabla u+\nabla u_{1}\right)\nabla \tilde{u}+c^{-1}\left(\alpha \theta +\theta_{1}\right)\tilde{\theta }\right\}\text{dx}.$$

$$\;=\int_{\Omega }\left\{\rho^{-1}\alpha v\tilde{v}+\rho^{-1}v_{1}\tilde{v}+\alpha \left(\lambda +2\mu \right)\nabla u\cdot \nabla \tilde{u}+\left(\lambda +2\mu \right)\nabla u_{1}\cdot \nabla \tilde{u}+\alpha c^{-1}\theta \tilde{\theta }+c^{-1}\theta_{1}\tilde{\theta }\right\}\text{dx}.$$

$$\begin{array}{cc} \; & =\int_{\Omega }\left\{\alpha \rho^{-1}v\tilde{v}+\alpha \left(\lambda +2\mu \right)\nabla u\cdot \nabla \tilde{u}+\alpha c^{-1}\theta \tilde{\theta }+\rho^{-1}v_{1}\tilde{v}+\left(\lambda +2\mu \right)\nabla u_{1}\cdot \nabla \tilde{u}+c^{-1}\theta_{1}\tilde{\theta }\right\}\text{dx}.\\ & =\alpha \int_{\Omega }\left\{\rho^{-1}v\tilde{v}+\left(\lambda +2\mu \right)\nabla u\cdot \nabla \tilde{u}+c^{-1}\theta \tilde{\theta }\right\}\text{dx}+\int_{\Omega }\left\{+\rho^{-1}v_{1}\tilde{v}+\left(\lambda +2\mu \right)\nabla u_{1}\cdot \nabla \tilde{u}+c^{-1}\theta_{1}\tilde{\theta }\right\}\text{dx}. \end{array}$$

$$\;=\alpha \langle \left(u,v,\theta \right),\left(\tilde{u},\tilde{v},\tilde{\theta }\right)\rangle +\langle \left(u_{1},v_{1},\theta_{1}\right),\left(\tilde{u},\tilde{v},\tilde{\theta }\right)\rangle .$$

$$\langle \left(u,v,\theta \right),\left(\tilde{u},\tilde{v},\tilde{\theta }\right)\rangle =\int_{\Omega }\left\{\rho^{-1}v\tilde{v}+\left(\lambda +2\mu \right)\nabla u.\nabla \tilde{u}+c^{-1}\theta \tilde{\theta }\right\}\text{dx}.$$

$$\;=\int_{\Omega }\left\{\rho^{-1}\tilde{v}v+\left(\lambda +2\mu \right)\nabla \tilde{u}.\nabla u+c^{-1}\tilde{\theta }\theta \right\}\text{dx}.$$

$$\;=\langle \left(\tilde{u},\tilde{v},\tilde{\theta }\right),\left(u,v,\theta \right)\rangle .$$

Then 〈.,.〉 is symmetric.

We show that 〈.,.〉 is positive definite.

$$\langle \left(u,v,\theta \right),\left(u,v,\theta \right)\rangle =\int_{\Omega }\left\{\rho^{-1}\text{vv}+\left(\lambda +2\mu \right)\nabla u.\nabla u+c^{-1}\theta \theta \right\}\text{dx}.$$

$$\;=\int_{\Omega }\left\{\rho^{-1}{\left|v\right|}^{2}+\left(\lambda +2\mu \right){\left|\nabla u\right|}^{2}+c^{-1}{\left|\theta \right|}^{2}\right\}\text{dx}.$$

$\rho^{-1}>0$, (λ+2μ)>0 and $c^{-1}>0$ then

$(\rho^{-1}{\left|v\right|}^{2}+\left(\lambda +2\mu \right){\left|\nabla u\right|}^{2}+c^{-1}{\left|\theta \right|}^{2})\geq 0$ on Ω.

So,

$$\int_{\Omega }\left\{\rho^{-1}{\left|v\right|}^{2}+\left(\lambda +2\mu \right){\left|\nabla u\right|}^{2}+c^{-1}{\left|\theta \right|}^{2}\right\}dx\geq 0.$$

We have 〈(u,v,θ),(u,v,θ)〉≥0. Moreover,

$$\langle \left(u,v,\theta \right),\left(u,v,\theta \right)\rangle =0\Leftrightarrow \int_{\Omega }\left\{\rho^{-1}{\left|v\right|}^{2}+\left(\lambda +2\mu \right){\left|\nabla u\right|}^{2}+c^{-1}{\left|\theta \right|}^{2}\right\}dx=0.$$

$$\langle \left(u,v,\theta \right),\left(u,v,\theta \right)\rangle =0\Leftrightarrow \rho^{-1}{\left|v\right|}^{2}+\left(\lambda +2\mu \right){\left|\nabla u\right|}^{2}+c^{-1}{\left|\theta \right|}^{2}=0.$$

〈(u,v,θ),(u,v,θ)〉=0⇔v=∇u=θ=0.

∇u=0 then u is constant with respect to x. u(t,0)=0, then u=0. We have

〈(u,v,θ),(u,v,θ)〉=0⇔u=v=θ=0.

Conclusion: 〈.,.〉 is inner product on H.

Proposition 3.2 *Equipped with the inner product 〈*.,.〉*defined by*(3.2)*is a Hilbert space*.

Proof. H equipped with the inner product 〈.,.〉 defined by (3.2) is a prehilbertian space by Proposition (3.1). We will show that H is complete.

Let ${\left(u^{n},v^{n},\theta^{n}\right)}_{n\geq 0}$ be a sequence of elements of H.

$$∥\left(u^{n},v^{n},\theta^{n}\right)-\left(u^{m},v^{m},\theta^{m}\right){∥}_{H}^{2}=\rho^{-1}∥v^{n}-v^{m}{∥}_{L^{2}\left(\Omega \right)}^{2}+\left(\lambda +2\mu \right)∥u^{n}-u^{m}{∥}_{H_{0}^{1}\left(\Omega \right)}^{2}+c^{-1}{∥\theta^{n}-\theta^{m}∥}_{L^{2}\left(\Omega \right)}^{2}.$$

Observe that therefore $\left(u^{n},v^{n},\theta^{n}\right)$ is a Cauchy sequence in H if and only if ${\left(u^{n}\right)}_{n}$ is a Cauchy sequence in $H^{2}\left(\Omega \right)\cap H_{0}^{1}\left(\Omega \right)$, ${\left(v^{n}\right)}_{n}$ is a Cauchy sequence in $L^{2}\left(\Omega \right)$ and ${\left(\theta^{n}\right)}_{n}$ is a Cauchy sequence in $L^{2}\left(\Omega \right)$. According to relation (3), the sequences $\left(u^{n}\right)$, $\left(v^{n}\right)$ and ($\theta^{n})$ are of Cauchy in $H^{2}\left(\Omega \right)\cap H_{0}^{1}\left(\Omega \right)$, $L^{2}\left(\Omega \right)$ and $L^{2}\left(\Omega \right)$ respectively.

$\left(u^{n}\right)$ is a Cauchy sequence in $H^{2}\left(\Omega \right)\cap H_{0}^{1}\left(\Omega \right)$ and since $H^{2}\left(\Omega \right)\cap H_{0}^{1}\left(\Omega \right)$ is complete, then there exists a rank $n_{1}$ from which $\left(u^{n}\right)$ converges to an element $l_{1}$ in $H^{2}\left(\Omega \right)\cap H_{0}^{1}\left(\Omega \right)$.

$\left(v^{n}\right)$ is a Cauchy sequence in $L^{2}\left(\Omega \right)$ and since $L^{2}\left(\Omega \right)$ is complete, then there exists a rank $n_{2}$ from which ($v^{n})$ converges to an element $l_{2}$ in $L^{2}\left(\Omega \right)$.

$\left(\theta^{n}\right)$ is a Cauchy sequence in $L^{2}\left(\Omega \right)$ and since $L^{2}\left(\Omega \right)$ is complete, then there exists a rank $n_{3}$ from which $\left(\theta^{n}\right)$ converges to an element $l_{3}$ in $L^{2}\left(\Omega \right)$.

Taking $n^{\prime }=max\left(n_{1},n_{2},n_{3}\right)$, ${\left(u^{n},v^{n},\theta^{n}\right)}_{n}$ converges to the triplet $\left(l_{1},l_{2},l_{3}\right)$ belonging to H from rank $n^{\prime }$.

Conclusion: H is a Hilbert space.

Let us define the operator A:D(A)⊆H→H by

$$D\left(A\right)=\left[H^{2}\left(\Omega \right)\cap H_{0}^{1}\left(\Omega \right)\right]\times \left[H^{2}\left(\Omega \right)\cap H_{0}^{1}\left(\Omega \right)\right]\times \left[H^{2}\left(\Omega \right)\cap H_{0}^{1}\left(\Omega \right)\right],$$

and

$$A\left(\begin{array}{c} u\\ \begin{array}{c} v\\ \theta \end{array} \end{array}\right)=\;\left(\begin{array}{c} \rho^{-1}v\\ \begin{array}{c} \left(\lambda +2\mu \right)\Delta u+m\nabla \theta \\ c^{-1}k\Delta \theta +c^{-1}\rho^{-1}\theta m\nabla v \end{array} \end{array}\right),$$

for each (u,v,θ)∈D(A). At this point, let us observe that the system (3.1) can be equivalently rewritten on the abstract form

(3.3) $$\left\{ \begin{array}{c} Z^{\prime }\left(t\right)=AZ\left(t\right),\;t\geq 0,\\ Z\left(0\right)=\xi ,\; \end{array}\right.$$

where $Z\left(t\right)\left(x\right)=\left(\begin{array}{c} u(t,x)\\ v(t,x)\\ \theta (t,x) \end{array}\right)$ and $\xi \left(x\right)=\left(\begin{array}{c} u_{0}\\ v_{0}\\ \theta_{0} \end{array}\right)$.

## Main results

### Existence and uniqueness of the solution of problem (3.3)

Theorem 4.1 *The operator -A is maximal monotone on H under the conditions (θ_m_ -*$ck^{-1}$*) < 0,*$(k^{-1}m+\lambda +2\mu )>0$*and*$\nabla u\cdot \nabla (v_{t}-v)\leq 0$.

Proof. We shall show that the operator I−A is surjective.

Let $(\tilde{u},\tilde{v},\tilde{\theta })\in H$ and (u,v,θ)∈D(A).

$$\left(I-A\right)\left(u,v,\theta \right)=\left(\tilde{u},\tilde{v},\tilde{\theta }\right)\Rightarrow$$

(4.1) $$\left\{ \begin{array}{c} u-\;\rho^{-1}v_{t\;}=\;\tilde{u},\;\\ v-\;\left(\lambda +2\mu \right)\Delta u+m\nabla \theta =\;\tilde{v},\;\\ \theta -\;c^{-1}k\Delta \theta +c^{-1}\rho^{-1}\theta m\nabla v=\;\tilde{\theta },\; \end{array}\right.$$

Since

$$-\;c^{-1}k\Delta \theta +c^{-1}\rho^{-1}\theta m\nabla v=\;\tilde{\theta },$$

we have

(4.2) $$\begin{array}{c} \Delta \theta -ck^{-1}\theta +k^{-1}\rho^{-1}\theta m\nabla v=-ck^{-1}\;\tilde{\theta }\\ \nabla v=\rho k\theta \Rightarrow \Delta \theta -ck^{-1}\theta +\theta m\theta =-ck^{-1}\;\tilde{\theta },\\ \Delta \theta +\left(\theta m-ck^{-1}\right)\theta =-ck^{-1}\tilde{\theta }\left(t,x\right). \end{array}$$

The characteristic equation of this last equation is $r^{2}+\theta m-ck^{-1}=0$ which has two real solutions ω and −ω with $\omega =\sqrt{-(\theta m-ck^{-1})}$ since $(\theta m-ck^{-1})<0$.

Thus the general solution to the inhomogenous 2nd order equation with constant coefficients is:

$$\theta \left(t,x\right)=C_{1}e^{\omega x}+C_{2}e^{-\omega x}+\int_{0}^{x}\left(C_{1}e^{\omega \left(x-y\right)}+C_{2}e^{-\omega \left(x-y\right)}\right)g\left(t,y\right)dy.$$

Besides $\theta \left(0,t\right)=0\Leftrightarrow C_{1}+C_{2}=0\Leftrightarrow C_{1}=-C_{2}$.

So $\theta \left(t,x\right)=C_{1}\left(e^{\omega x}-e^{-\omega x}+C_{1}\int_{0}^{x}(e^{\omega (x-y)}-e^{-\omega (x-y)}\right)g\left(t,y\right)dy,$

$with\;g\left(t,x\right)=\;-ck^{-1}\tilde{\theta }\left(t,x\right)$. Then

$$\theta \left(t,x\right)=2C_{1}sh\left(\omega x\right)+2C_{1}\int_{0}^{x}sh\left(\omega \left(x-y\right)\right)g\left(t,y\right)dy.$$

Since $u-\rho^{-1}v_{t}=\tilde{u}$ we have

$$u-\rho^{-1}\left(\lambda +2\mu \right)\Delta u-\rho^{-1}m\nabla \theta =\tilde{u}.$$

$$\nabla v=\rho k\theta \Rightarrow \theta =\rho^{-1}k^{-1}\nabla v.$$

$$\nabla v=\rho k\theta \Rightarrow \nabla \theta =\rho^{-1}k^{-1}\Delta v.$$

v=ρu⇒Δv=ρΔu.

Then $\nabla \theta =k^{-1}\Delta u.$

$$u-\rho^{-1}\left(\lambda +2\mu \right)\Delta u-\rho^{-1}m\nabla \theta =\tilde{u}.$$

$$u-\rho^{-1}\left(\lambda +2\mu \right)\Delta u-\rho^{-1}k^{-1}m\Delta u=\tilde{u}.$$

(4.3) $$\begin{array}{c} u-\rho^{-1}\left(k^{-1}m+\lambda +2\mu \right)\Delta u=f\left(t,x\right) \end{array},$$

with

$$f\left(t,x\right)=\tilde{u}\left(t,x\right).$$

Thus we obtain a linear differential equation of second order with second member. The homogeneous equation has characteristic equation

$$-\rho^{-1}\left(k^{-1}m+\lambda +2\mu \right)r^{2}+1=0.$$

Since $(k^{-1}m+\lambda +2\mu )>0$, then the characteristic equation admits two real solutions $\omega_{1}$ and $-\omega_{1}$ with $\omega_{1}=\sqrt{\rho {\left(k^{-1}m+\lambda +2\mu \right)}^{-1}}$ . Consequently the differential equation (4.2) has a unique solution u gived by:

$$u\left(t,x\right)=C_{1}e^{\omega_{1}x}+C_{2}e^{-\omega_{1}x}+\int_{0}^{x}\left(C_{1}e^{\omega_{1}\left(x-y\right)}+C_{2}e^{-\omega_{1}\left(x-y\right)}\right)f\left(t,y\right)dy.$$

Where $C_{1}$ and $C_{2}$ are real constants. $u\left(0,t\right)=0\Leftrightarrow C_{1}+C_{2}=0\Leftrightarrow C_{1}=-C_{2}$.

$$u\left(t,x\right)=C_{1}\left(e^{\omega_{1}x}-e^{-\omega_{1}x}+C_{1}\int_{0}^{x}(e^{\omega_{1}\left(x-y\right)}-e^{-\omega_{1}\left(x-y\right)}\right)f\left(t,y\right)\text{dy}.$$

$$u\left(t,x\right)=2C_{1}\text{sh}\left(\omega_{1}x\right)+2C_{1}\int_{0}^{x}\text{sh}\left(\omega_{1}\left(x-y\right)\right)f\left(t,y\right)\text{dy}.$$

So, for any triplet $(\tilde{u},\tilde{v},\tilde{\theta })\in H$, there exists a triplet (u,v,θ)∈D(A) such that

$$\left(I-A\right)\left(u,v,\theta \right)=\left(\tilde{u},\tilde{v},\tilde{\theta }\right),$$

that is to say I−A is surjective and Im(I−A)=H.

(4.4) $$\langle \left(u,v,\theta \right),\left(\tilde{u},\tilde{v},\tilde{\theta }\right)\rangle =\int_{\Omega }\left\{\rho^{-1}v\tilde{v}+\left(\lambda +2\mu \right)\nabla u\cdot \nabla \tilde{u}+c^{-1}\theta \tilde{\theta }\right\}dx.$$

$$A\left(\begin{array}{c} u\\ \begin{array}{c} v\\ \theta \end{array} \end{array}\right)=\left(\begin{array}{c} \rho^{-1}v_{t}\\ \begin{array}{c} \left(\lambda +2\mu \right)\Delta u+m\nabla \theta \\ c^{-1}k\Delta \theta +c^{-1}\rho^{-1}\theta m\nabla v \end{array} \end{array}\right),$$

$$\begin{array}{c} \langle A\left(u,v,\theta \right),\left(u,v,\theta \right)\rangle =\int_{\Omega }\left\{\rho^{-1}\left[\left(\lambda +2\mu \right)\Delta u+m\nabla \theta \right]v+\rho^{-1}\left[\left(\lambda +2\mu \right)\nabla u\cdot \nabla v_{t}\right]\right\}\\ \;+c^{-2}k\theta .\Delta \theta +c^{-2}\rho^{-1}\theta m\theta .\nabla vdx. \end{array}$$

(4.5) $$\begin{array}{c} <A\left(u,v,\theta \right),\left(u,v,\theta \right)\geq \rho^{-1}\int_{\Omega }(\lambda +2\mu )v\cdot \Delta udx+\rho^{-1}m\int_{\Omega }v\cdot \nabla \theta dx+\rho^{-1}\int_{\Omega }\left(\lambda +2\mu \right)\nabla u\cdot \nabla v_{t}dx\\ \;+c^{-2}k\int_{\Omega }\theta \cdot \Delta \theta dx\;+c^{-2}\rho^{-1}\theta m\int_{\Omega }\theta \cdot \nabla vdx. \end{array}$$

(4.6) $$\rho^{-1}\int_{\Omega }\left(\lambda +2\mu \right)v\cdot \Delta udx=\rho^{-1}{\left[\left(\lambda +2\mu \right)v\cdot \nabla u\right]}_{\partial \Omega }-\rho^{-1}\int_{\Omega }\left(\lambda +2\mu \right)\nabla u\cdot \nabla vdx.$$

(4.7) $$\rho^{-1}\int_{\Omega }\left(\lambda +2\mu \right)v\cdot \Delta udx=-\rho^{-1}\int_{\Omega }\left(\lambda +2\mu \right)\nabla u\cdot \nabla vdx.$$

(4.8) $$\rho^{-1}m\int_{\Omega }v\cdot \nabla \theta dx=\rho^{-1}m{\left[\theta \cdot v\right]}_{\partial \Omega }-\rho^{-1}m\int_{\Omega }\theta \cdot \nabla \theta vdx.$$

(4.9) $$\rho^{-1}m\int_{\Omega }v\cdot \nabla \theta dx=-\rho^{-1}m\int_{\Omega }\theta \cdot \nabla vdx.$$

(4.10) $$c^{-2}k\int_{\Omega }\theta \cdot \Delta \theta dx=c^{-2}k{\left[\theta \cdot \nabla \theta \right]}_{\partial \Omega }-c^{-2}k\int_{\Omega }\nabla \theta \cdot \nabla \theta dx.$$

(4.11) $$c^{-2}k\int_{\Omega }\theta \cdot \Delta \theta dx=-c^{-2}k\int_{\Omega }\nabla \theta \cdot \nabla \theta dx$$

(4.12) $$\begin{array}{cc} & \begin{array}{c} <A\left(u,v,\Omega \right),\left(u,v,\theta \right)\geq -\rho^{-1}\left(\lambda +2\mu \right)\int_{\Omega }\nabla u\cdot \nabla vdx-\rho^{-1}m\int_{\Omega }\theta \cdot \nabla vdx \end{array}\\ & \;+\rho^{-1}\left(\lambda +2\mu \right)\int_{\Omega }\nabla u\cdot \nabla v_{t}dx-c^{-2}k\int_{\Omega }\nabla \theta \cdot \nabla \theta dx+\;-c^{-2}\rho^{-1}\theta m\int_{\Omega }\theta \cdot \nabla vdx. \end{array}$$

$$\frac{\theta }{c^{2}}=1\;\Rightarrow \;c^{-2}\rho^{-1}\theta m=m\rho^{-1},$$

(4.13) $$\begin{array}{cc} & <A\left(u,v,\theta \right),\left(u,v,\theta \right)\geq -\rho^{-1}\left(\lambda +2\mu \right)\int_{\Omega }\nabla u\cdot \nabla vdx-\rho^{-1}m\int_{\Omega }\theta \cdot \nabla vdx\\ & +\;\rho^{-1}\left(\lambda +2\mu \right)\int_{\Omega }\nabla u\cdot \nabla v_{t}dx-c^{-2}k\int_{\Omega }\nabla \theta \cdot \nabla \theta dx+\rho^{-1}m\int_{\Omega }\theta \cdot \nabla vdx. \end{array}$$

then

(4.14) $$<A\left(u,v,\theta \right),\left(u,v,\theta \right)\geq -c^{-2}k\int_{\Omega }\nabla \theta \cdot \nabla \theta dx+\rho^{-1}\left(\lambda +2\mu \right)\int_{\Omega }\nabla u\cdot \nabla \left(v_{t}-v\right)dx.$$

(4.15) $$\langle A\left(u,v,\theta \right),\left(u,v,\theta \right)\rangle =-c^{-2}k\int_{\Omega }∥\nabla \theta {∥}^{2}dx+\rho^{-1}\left(\lambda +2\mu \right)\int_{\Omega }\nabla u\cdot \nabla \left(v_{t}-v\right)dx.$$

Since k*θ*^−1^> 0 and $\nabla u\cdot \nabla (v_{t}-v)\leq 0$, then

〈A(u,v,θ),(u,v,θ)〉≤0.

So,

〈−A(u,v,θ),(u,v,θ)〉=−〈A(u,v,θ),(u,v,θ)〉≥0.

Consequently, the operator −A is monotone.

The operator −A is monotone and Im(I−A)=H, therefore the operator −A is maximal monotone. Using the theorem 2.2, the problem (3.3) admits a unique solution Z(t)(x)=T(t)ξ(x) for all ξ∈D(A) where ${\left(T(t)\right)}_{t\geq 0}$ is the $C_{0}$-semi-group of contractions generated by A.

### Stability of the solution of the initial value problem

We shall show that the system is uniformly exponentially stable under the condition m=0.

Indeed, for m=0, we have the following system

(4.16) $$\left\{ \begin{array}{cc} u_{t}=\rho^{-1}v_{t} & \left(t,x\right)\in R_{+}\times \Omega ,\\ v_{t}=\left(\lambda +2\mu \right)\Delta u & \left(t,x\right)\in R_{+}\times \Omega ,\\ \theta_{t}\;=c^{-1}k\Delta \theta & \left(t,x\right)\in R_{+}\times \Omega ,\\ u\left(t,x\right)=\theta \left(t,x\right)=0 & \left(t,x\right)\in R_{+}\times \partial \Omega ,\\ u\left(0,x\right)=u_{0}\left(x\right) & x\in \;\Omega ,\\ v_{0}\left(x\right)=v\left(0,x\right) & x\in \;\Omega ,\\ \theta \left(0,x\right)=\theta_{0}\left(x\right) & x\in \;\Omega , \end{array}\right.$$

Proposition 4.2 $$\int_{0}^{\infty }∥T\left(t\right)\xi {∥}^{2}dt<\infty ,$$

for every ξ∈H.

Let ${\left(\lambda_{n}\right)}_{n\geq 1}$ be an the increasing sequence of eigenvalues of the operator Δ. The eigenvectors ${\left(e_{n}\right)}_{n}$ associated with $\lambda_{n}\left(-\Delta e_{n}=\lambda_{n}e_{n}\right)$ form a Hilbertian basis. Let ${\left(T_{1}\left(t\right)\right)}_{t\geq 0}$ be the $C_{0}$-semi-group generated by the operator Δ with $D\left(\Delta \right)=H^{2}\left(\Omega \right)\cap H_{0}^{1}\left(\Omega \right)$ and Ω=[0,π]. We pose $f=\sum_{n=1}^{\infty }\left(f,e_{n}\right)e_{n}$.

We have

$$T_{1}\left(t\right)f=\sum_{n=1}^{\infty }\left(f,e_{n}\right)e_{n}e^{-n^{2}t}.$$

Lemma 4.3 *Let*${\left(S(t)\right)}_{t\geq 0}$*be the*$C_{0}$*-semi-group generated by the operator A. Then for all*α>0*,*αA*generates*${\left(S(\alpha t)\right)}_{t\geq 0}$.

Since the constants $c^{-1}k$ and $\rho^{-1}\left(\lambda +2\mu \right)$ are strictly positive, then $c^{-1}k\Delta$ and $\rho^{-1}\left(\lambda +2\mu \right)\Delta$ generate ${\left(T_{1}\left(c^{-1}kt\right)\right)}_{t\geq 0}$ and ${\left(T_{1}\left(\rho^{-1}\left(\lambda +2\mu \right)t\right)\right)}_{t\geq 0}$ respectively.

Since $T_{1}\left(t\right)f=\sum_{n=1}^{\infty }\left(f,e_{n}\right)e_{n}e^{-n^{2}t}$, then

$$T_{1}\left(c^{-1}kt\right)e_{n}=\sum_{n=1}^{\infty }e^{-c^{-1}kn^{2}t}e_{n}$$

and

$$T_{1}\left(\rho^{-1}\left(\lambda +2\mu \right)t\right)=\sum_{n=1}^{\infty }e^{-\rho^{-1}\left(\lambda +2\mu \right)n^{2}t}e_{n}.$$

The equation $\theta_{t}=c^{-1}k\Delta \theta$ with θ(0,t)=θ(π,t)=0 and $\theta \left(x,0\right)=\theta_{0}\left(x\right)$ admits a unique solution defined by

$$\theta \left(t,x\right)=T_{1}\left(c^{-1}kt\right)\theta_{0}\left(x\right).$$

The equation $u_{t}=\rho^{-1}\left(\lambda +2\mu \right)\Delta u$ with u(0,t)=u(π,t)=0 and $u\left(x,0\right)=u_{0}\left(x\right)$ admits a unique solution defined by

$$u\left(t,x\right)=T_{1}\left(\rho^{-1}\left(\lambda +2\mu \right)t\right)u_{0}\left(x\right).$$

$$v_{t}\left(t,x\right)=\rho u_{t}\left(t,x\right)\Rightarrow v\left(t,x\right)=\rho u\left(t,x\right)+c.$$

v(t,0)=u(t,0)=0⇒c=0.

Thus

$$v\left(t,x\right)=\rho u\left(t,x\right)=T_{1}\left(\rho^{-1}\left(\lambda +2\mu \right)t\right)\rho u_{0}\left(x\right)=T_{1}\left(\rho^{-1}\left(\lambda +2\mu \right)t\right)v_{0}\left(x\right),$$

with $v_{0}\left(x\right)=\rho u_{0}\left(x\right).$

It follows that:

T(t)ξ(x)=(u(t,x),v(t,x),θ(t,x)).

$$∥T\left(t\right)\xi \left(x\right){∥}_{H}^{2}=\langle \left(u\left(t,x\right),v\left(t,x\right),\theta \left(t,x\right)\right),\left(u\left(t,x\right),v\left(t,x\right),\theta \left(t,x\right)\right)\rangle .$$

$$\int_{\Omega }\left\{\rho^{-1}{\left|v\left(t,x\right)\right|}^{2}+\left(\lambda +2\mu \right){\left|\nabla u\left(t,x\right)\right|}^{2}+c^{-1}{\left|\theta \left(t,x\right)\right|}^{2}\right\}\text{dx}.$$

$$\;=\rho^{-1}\int_{\Omega }{\left|v\left(t,x\right)\right|}^{2}\text{dx}+\left(\lambda +2\mu \right)\int_{\Omega }{\left|\nabla u\left(t,x\right)\right|}^{2}\text{dx}+c^{-1}\int_{\Omega }{\left|\theta \left(t,x\right)\right|}^{2}\text{dx}.$$

$$\;=\rho^{-1}\int_{\Omega }{\left|T_{1}\left(\rho^{-1}\left(\lambda +2\mu \right)t\right)v_{0}\left(x\right)\right|}^{2}\text{dx}+\left(\lambda +2\mu \right)\int_{\Omega }{\left|T_{1}\left(\rho^{-1}\left(\lambda +2\mu \right)t\right)\nabla u_{0}\left(x\right)\right|}^{2}\text{dx}+c^{-1}\int_{\Omega }{\left|T_{1}\left(c^{-1}\text{kt}\right)\theta_{0}\left(x\right)\right|}^{2}\text{dx}.$$

$$\;\leq \rho^{-1}{\left|T_{1}\left(\rho^{-1}\left(\lambda +2\mu \right)t\right)\right|}^{2}\int_{\Omega }{\left|v_{0}\left(x\right)\right|}^{2}\text{dx}+\left(\lambda +2\mu \right){\left|T_{1}\left(\rho^{-1}\left(\lambda +2\mu \right)t\right)\right|}^{2}\int_{\Omega }{\left|\nabla u_{0}\left(x\right)\right|}^{2}\text{dx}+c^{-1}{\left|T_{1}\left(c^{-1}\text{kt}\right)\right|}^{2}\int_{\Omega }{\left|\theta_{0}\left(x\right)\right|}^{2}\text{dx}.$$

$$\;\leq \rho^{-1}\sum_{n=1}^{\infty }e^{-2\rho^{-1}\left(\lambda +2\mu \right)n^{2}t}∥v_{0}{∥}_{L^{2}\left(\Omega \right)}^{2}+\left(\lambda +2\mu \right)\sum_{n=1}^{\infty }e^{-2\rho^{-1}\left(\lambda +2\mu \right)n^{2}t}∥u_{0}{∥}_{H_{0}^{1}\left(\Omega \right)}^{2}+c^{-1}\sum_{n=1}^{\infty }e^{-2c^{-1}kn^{2}t}{∥\theta_{0}∥}_{L^{2}\left(\Omega \right)}^{2}.$$

We have $H=\left(H^{2}\left(\Omega \right)\cap H_{0}^{1}\left(\Omega \right)\right)\;\times L^{2}\left(\Omega \right)\;\times L^{2}\left(\Omega \right)$ so for all ξ∈H, $u_{0}\in H^{2}\left(\Omega \right)\cap H_{0}^{1}\left(\Omega \right),$ $v_{0}\in L^{2}\left(\Omega \right),$ and $\theta_{0}\in L^{2}\left(\Omega \right)$. Since Ω a bounded open, then there exists α>0, β>0, γ>0 such that:

$$∥v_{0}{∥}_{L^{2}\left(\Omega \right)}^{2}<\alpha ,∥u_{0}{∥}_{H_{0}^{1}\left(\Omega \right)}^{2}<\beta \;\text{and}\;{∥\theta_{0}∥}_{L^{2}\left(\Omega \right)}^{2}<\gamma .$$

$$\rho^{-1}\sum_{n=1}^{\infty }e^{-2\rho^{-1}\left(\lambda +2\mu \right)n^{2}t}{∥v_{0}∥}_{L^{2}\left(\Omega \right)}^{2}<\alpha \rho^{-1}\sum_{n=1}^{\infty }e^{-2\rho^{-1}\left(\lambda +2\mu \right)n^{2}t}.$$

$$\left(\lambda +2\mu \right)\sum_{n=1}^{\infty }e^{-2\rho^{-1}\left(\lambda +2\mu \right)n^{2}t}{∥u_{0}∥}_{H_{0}^{1}\left(\Omega \right)}^{2}<\beta \left(\lambda +2\mu \right)\sum_{n=1}^{\infty }e^{-2\rho^{-1}\left(\lambda +2\mu \right)n^{2}t}.$$

$$c^{-1}\sum_{n=1}^{\infty }e^{-2c^{-1}kn^{2}t}{∥\theta_{0}∥}_{L^{2}\left(\Omega \right)}^{2}<\gamma c^{-1}\sum_{n=1}^{\infty }e^{-2c^{-1}kn^{2}t}.$$

We have:

$$∥T\left(t\right){\xi ∥}_{H}^{2}\leq \alpha \rho^{-1}\sum_{n=1}^{\infty }e^{-2\rho^{-1}\left(\lambda +2\mu \right)n^{2}t}+\beta \left(\lambda +2\mu \right)\sum_{n=1}^{\infty }e^{-2\rho^{-1}\left(\lambda +2\mu \right)n^{2}t}+\gamma c^{-1}\sum_{n=1}^{\infty }e^{-2c^{-1}kn^{2}t}.$$

$$\int_{0}^{\infty }∥T\left(t\right){\xi ∥}_{H}^{2}\text{dt}=\underset{\epsilon \to +\infty }{\text{lim}}\int_{0}^{\epsilon }{∥T\left(t\right)\xi ∥}_{H}^{2}\text{dt}.$$

$$\underset{\epsilon \to +\infty }{\text{lim}}\left({\left[\frac{-\alpha \rho^{-1}}{2\rho^{-1}\left(\lambda +2\mu \right)n^{2}}e^{-2\rho^{-1}\left(\lambda +2\mu \right)n^{2}t}\right]}_{0}^{\epsilon }\right)=\underset{\epsilon \to +\infty }{\text{lim}}\left(\frac{-\alpha \rho^{-1}}{2\rho^{-1}\left(\lambda +2\mu \right)n^{2}}e^{-2\rho^{-1}\left(\lambda +2\mu \right)n^{2}\epsilon }+\frac{\alpha }{2\left(\lambda +2\mu \right)n^{2}}\right).$$

$$\underset{\epsilon \to +\infty }{\text{lim}}\left({\left[\frac{-\alpha \rho^{-1}}{2\rho^{-1}\left(\lambda +2\mu \right)n^{2}}e^{-2\rho^{-1}\left(\lambda +2\mu \right)n^{2}t}\right]}_{0}^{\epsilon }\right)=\frac{\alpha }{2\left(\lambda +2\mu \right)n^{2}}.$$

$$\underset{\epsilon \to +\infty }{\text{lim}}\left({\left[\frac{-\beta \left(\lambda +2\mu \right)}{2\rho^{-1}\left(\lambda +2\mu \right)n^{2}}e^{-2\rho^{-1}\left(\lambda +2\mu \right)n^{2}t}\right]}_{0}^{\epsilon }\right)=\underset{\epsilon \to +\infty }{\text{lim}}\left(-\frac{\beta \left(\lambda +2\mu \right)}{2\rho^{-1}\left(\lambda +2\mu \right)n^{2}}e^{-2\rho^{-1}\left(\lambda +2\mu \right)n^{2}\epsilon }+\frac{\beta \rho }{2n^{2}}\right).$$

$$=\frac{\beta \rho }{2n^{2}}.$$

$$\underset{\epsilon \to +\infty }{\text{lim}}\left({\left[\frac{-\gamma c^{-1}}{2c^{-1}kn^{2}}e^{-2c^{-1}kn^{2}t}\right]}_{0}^{\epsilon }\right)=\underset{\epsilon \to +\infty }{\text{lim}}\left(-\frac{\gamma c^{-1}}{2c^{-1}kn^{2}}e^{-2c^{-1}kn^{2}\epsilon }+\frac{\gamma }{2kn^{2}}\right).$$

$$=\frac{\gamma }{2kn^{2}}.$$

$$\int_{0}^{\infty }{∥T\left(t\right)\xi ∥}_{H}^{2}\text{dt}\leq \sum_{n=1}^{\infty }\left(\frac{\alpha }{2\left(\lambda +2\mu \right)n^{2}}+\frac{\beta \rho }{2n^{2}}+\frac{\gamma }{2kn^{2}}\right).$$

$$\int_{0}^{\infty }{∥T\left(t\right)\xi ∥}_{H}^{2}\text{dt}<\infty .$$

for all ξ∈H. ${\left(T(t)\right)}_{t\geq 0}$ is uniformly exponentially stable according the Proposition 2.7.

## Application examples in thermomechanical coupling

During mechanical loading of an elastic body, some work is done due to straining. This energy dissipates as heat induces a temperature field within the material. So, in Fourier heat conduction equation, this internal heat source should be appropriately included for accurately computing the temperature field. The coupling between the temperature and strain fields also helps in determining the temperature field due to time-varying forces and also accounts for the influence of temperature on the velocity of propagation of elastic waves. Only in stationary temperature fields, this coupling term may be neglected [[6], [7], [8], [20]].

Stress, strain, and temperature relations in isotropic and homogeneous theroelastic solids (Duhamel–Neumann relations) are

(5.1) $$\sigma_{ij}=\left(\lambda \mu_{i,i}-\beta \theta \right)\delta_{i,i}+2\mu e_{ij},\left(i,j=1,2,3\right)$$

where λ and μ are Lamè’s constants, $\beta =\left(3\lambda +2\mu \right)\alpha_{i}$, $\alpha_{i}$ is the coefficient of linear thermal expansion of the material, $\sigma_{ij}$ is the stress tensor, θ is the increase in temperature above reference temperature $T_{0}$, $e=u_{i,i}=$ dilatation, $e_{ij}$ are given by $e_{ij}=\frac{1}{2}\left(u_{i,j}+u_{j,i}\right)$ with $e_{i,j}=e_{j,i0}$. These equations are to be supplemented by classical Fourier's law connecting heat flux vector $\bar{q}$ with temperature gradien $\bar{\nabla }\theta$ by the equation

(5.2) $$\bar{q}=-k\bar{\nabla }\theta \;\text{or}\;q_{i}=-k\theta_{i},i=1,2,3$$

i.e., heat flux vector is the instantaneous result of a temperature gradient and k is the thermal conductivity. When coupling of strain and temperature field is taken into account, the principal of local energy balance gives

$$-q_{i,i}+\rho R=\rho c_{v}\dot{\theta }+\beta T_{0}\dot{e}$$

ie

(5.3) $$-\bar{\nabla }\bar{q}+\rho R=\rho c_{v}\dot{\theta }+\beta T_{0}\dot{e}$$

where ρ is the mass density, $c_{v}$ is the specific heat of the solid at constant volume, and t is the time. Then, coupled heat conduction equation by elimination of $q_{i}$ is

(5.4) $$k\Delta \theta +\rho R=\rho c_{v}\dot{\theta }+\theta_{0}\beta {\dot{u}}_{k;k}$$

The term $T_{0}$ brings to consider coupling between strain and temperature. Again the principle of balance of linear momentum leads to the stress *equations* of motion of the linearized form

(5.5) $$\sigma_{ij,j}+\rho f_{i}=\rho {\ddot{u}}_{i};i,j=1,2,3$$

where $f_{i}$ ’s are the components of external body force vector per unit mass.

Eqs. (5.1), and (5.5) lead to the displacement equations of motion

(5.6) $$\left(\lambda +2\mu \right)\Delta u_{i,i}+\rho f_{i}-\beta \theta_{i}=\rho {\ddot{u}}_{i}$$

Eq. (5.4) is a parabolic-type equation whereas Eq. (5.6) is of hyperbolic type. The Eq. (5.4) is due to Biot, and it is concerned with the interaction of the thermal field and elastic deformation such that the two fields are coupled. A direct consequence of Eq. (5.4) based on classical Fourier's law is that if the material is subjected to a thermal disturbance, the effect of the disturbances in both temperature and displacement fields will be at distance infinitely far from the heat source since the two fields are coupled. This amounts to saying that the thermal signals propagate with infinite speed. Consequently, the result is physically unrealistic, particularly for initial value problems and very short time intervals, and all classical thermodynamical theories suffer from this drawback [[9], [10], [11], [12]].

## Conclusion

This article deals with a generalization with λ+2μ > 0, ck > 0 of the model studied in [1] with $v=\rho u_{t}$, λ+2μ=1, ck=1 and div[σ(u)]=Δu. We have obtained in our work, the results of existence, uniqueness and stability of the solution which coincide with the conditions, λ+2μ=1 et ck=1 of Amar Herminna, Abdoulaye Sene and Serge Nicaise.

## Declaration of Competing Interest

The authors declare that they have no known competing financial interests or personal relationships that could have appeared to influence the work reported in this paper.

## Data Availability

No data was used for the research described in the article.
