# Corrigendum to “Quantitative and stability study of the evolution of a thermoelastic body” [MethodsX 10 (2023) 101983]

**DOI:** 10.1016/j.mex.2024.103016

**Published:** 2024-11-08

**Authors:** Pascal H. Zinsou, Guy Degla, Khalil Ezzinbi

**Affiliations:** a Institut De Mathematiques Et De Sciences Physiques (IMSP), 01 BP 613 PORTO-NOVO, Benin; b Universite Cardi Ayyad, Facultedes Sciences Semlalia, Departement De Mathematiques, B.P.2390, Marrakesh, Morocco

**Keywords:** Thermoelasticity, Operator maximal monotone, Semi-group of contraction

## Abstract

We prove the existence and uniqueness of a solution to a system of equations describing the evolution of a linear thermoelastic body by using a semi-group method. Moreover, the uniform exponential stability of the solution is shown in a particular case.

• With respect to the existence and uniqueness of the solution, we have defined a linear operator which generates a contraction semigroup and show that it is monotone maximal.
• With respect to the stability of the system, we have computed explicitly the expression of the solution of the system and show that the semigroup is uniformly exponentially stable in a particular case.

The authors regret.

## Introduction

In this paper, we are interested in a system of equations describing the evolution of a linear thermoelastic body:

(1.1) $$\left\{ \begin{array}{c} u_{t}=\rho^{-1}v_{\;}\;\;\;\;\;\left(t,x\right)\in R_{+}\;\times \;\Omega \\ v_{t}=\left(\lambda +2\mu \right)\Delta u+m\nabla \theta \;\;\left(t,x\right)\in R_{+}\;\times \;\Omega \\ \;\theta_{t}=c^{-1}k\Delta \theta +c^{-1}\rho^{-1}m\;\Theta \;\nabla v\;\left(t,x\right)\in R_{+}\;\times \;\Omega \\ u\left(t,x\right)=\theta \left(t,x\right)=0\;\;\;\;\;\;\left(t,x\right)\in R_{+}\;\times \;\partial \Omega \\ u\left(0,x\right)=u_{0}\left(x\right)\;\;\;\;x\;\in \;\Omega \\ v_{0}\left(x\right)=v\left(0,x\right)\;\;\;\;x\;\in \;\Omega \\ \theta \left(0,x\right)=\theta_{0}\left(x\right)\;\;\;\;x\;\in \;\Omega \end{array}\right.$$

where ρ>0 is the density of the body that *ρ* is constant, Θ ˃ 0 the referential temperature, and c>0, k>0, m, λ, μ are constants. Ω is a nonempty, open and bounded subset in $R^{n}.$ u(t,x) the displacement, v(t,x) the momentum and θ(t,x) the temperature. Several mathematical models that come from physics (thermoelasticity motion) lead to the study of partial differential equations (PDEs) and sometimes evolution equations allowing mathematicians to describe the behavior of a quantity that depends on several variables. Thermoelasticity is the elasticity of bodies resulting from an increase in temperature. Thermo-elasticity is the appropriate model for the explanation of the decay of the amplitude of free vibrations of some elastic bodies. Since then, a wide variety of results and applications have been obtained in many different fields. For instance, in [[1], [2], [3], [4]] synthetic tissues which mimic human bones are investigated, while in [[5], [6], [7], [8]] cardiological tissues are considered. Besides a model of apples regarded as thermoelastic bodies is studied in [[9], [10], [11], [12], [13], [14], [15], [19], [20]]. The present investigation concerns thermoelastic bodies and their thermic behaviour aiming to widen the range of applicable cases the theory can be applied to. In [16], Amar Herminna, Abdoulaye Sene and Serge Nicaise studied the existence, uniqueness and stability of a solution of this system with λ+2μ=1, ck=1 and divσ(u)=Δu. In this work, we have shown the existence and uniqueness of a solution of this system in a general case and stability in a particular case by using a semi-group method.

## Preliminaries

Definition 2.1 [17] Let X be a Hilbert space. Λ:D(Λ)⊆X→X a linear operator. We say that Λ is monotone if 〈Λx,x〉≥0 for all x∈D(Λ). Λ is maximal monotone if Λ is monotone and Im(I+Λ)=X.

The following theorem is an approach of Lumer Philippe's theorem very used to show the existence and the uniqueness of the solution of an evolution system.

Theorem 2.2 [17] Let X be a real Hilbert space. Λ:D(Λ)⊆X→X a linear operator. If −Λ is maximal monotone of X, then Λ is the infinitesimal generator of a $C_{0}$-semi-group of contractions ${\left(T(t)\right)}_{t\geq 0}$ on X.

We will then present some definitions and theorems of the stability of an evolution system.

Definition 2.3 [18] We define the growth rate of the semi-group ${\left(T(t)\right)}_{t\geq 0}$ by $w_{0}\left(T\right)=inf\left\{w\in R;\exists M\geq 1/∥T\left(t\right)∥\leq Me^{wt},t\geq 0\right\}.$

Definition 2.4 [18] We call spectral bound of an operator Λ∈£(X), the number noted S(Λ) defined by (Λ)=sup{Re(λ);λ∈σ(Λ)}.

Definition 2.5 [[13], [17]] Let be an infinitesimal generator of a $C_{0}$-semi group ${\left(T(t)\right)}_{t\geq 0}$ generated by A. Then we have:

$$\underset{t>0}{\text{inf}}\frac{log∥T\left(t\right)∥}{t}=\underset{t\to +\infty }{\text{lim}}\frac{log∥T\left(t\right)∥}{t}\leq \frac{1}{t_{0}}log\left(r\left(T\left(t_{0}\right)\right)\right)$$

$$-\infty \leq s\left(A\right)\leq w_{0}\left(A\right)<+\infty$$

for each $t_{0}>0$.

Definition 2.6 [18] Let ${\left(T(t)\right)}_{t\geq 0}$ be a $C_{0}$-semi group on a Banach space X. ${\left(T(t)\right)}_{t\geq 0}$ is exponentially stable if and only if,$\;w_{0}\left(T\right)<0$.

Proposition 2.7 [20] Let be ${\left(T(t)\right)}_{t\geq 0}$ a $C_{0}$-semi group on a Banach space X. ${\left(T(t)\right)}_{t\geq 0}$ is uniformly exponentially stable if and only if, for some p, 1≤p<∞

$$\int_{0}^{\infty }T\left(t\right)\xi^{p}dt<\infty$$

for every ξ∈X.

## Mathematical model

Let C be a homogeneous body having as referential configuration a nonempty, open and bounded subset Ω in R whose boundary Γ is of class $C^{1}$. The state of the body at the time t∈R, is characterized by two vector fields: the displacement u(t,x), the momentum v(t,x), and a scalar fields: the temperature θ(t,x). The system of equations describing the evolution of these three fields is:

(3.1) $$\left\{ \begin{array}{c} u_{t}=\rho^{-1}v_{\;}\;\;\;\;\left(t,x\right)\in R_{+}\;\times \;\Omega \\ v_{t}=\left(\lambda +2\mu \right)\Delta u+m\nabla \theta \;\;\;\left(t,x\right)\in R_{+}\;\times \;\Omega \\ \;\theta_{t}=c^{-1}k\Delta \theta +c^{-1}\rho^{-1}m\;\Theta \;\;\nabla v\;\left(t,x\right)\in R_{+}\;\times \;\Omega \\ u\left(t,x\right)=\theta \left(t,x\right)=0\;\;\;\left(t,x\right)\in R_{+}\;\times \;\partial \Omega \\ u\left(0,x\right)=u_{0}\left(x\right)\;\;\;\;x\;\in \;\Omega \\ v_{0}\left(x\right)=v\left(0,x\right)\;\;\;\;x\;\in \;\Omega \\ \theta \left(0,x\right)=\theta_{0}\left(x\right)\;\;\;\;x\;\in \;\Omega \end{array}\right.$$

where ρ>0 is the density of the body specify that *ρ* is constant Θ>0 the referential temperature, and c>0, k>0, m, λ, μ are constants which characterized the thermoelastic properties of the body with $C^{2}=\Theta$ and ∇v=ρkθ with $\nabla u=\frac{\partial }{\partial x}u$ and $\Delta u=\frac{\partial^{2}}{\partial x^{2}}u$. Composite materials used as structural elements in high-tech fields (aerospace industry, automotive industry...) are subjected in many cases to thermal stresses (turbojet $1000^{c}$, supersonic combustion $1750^{c}$, missile cone...) The components of these composites do not expand in the same way. This difference in expansion coefficients can lead to plasticization or total failure. This justifies the need to determine the thermoelastic behavior. So, the present system is a generalization of the evolution of bodies in motion of thermoelasticity. We begin by rewriting the system (3.1) under the form of an abstract Cauchy problem in a suitably chosen Hilbert space. More precisely, let

$$H=\left[H_{0}^{1}\left(\Omega \right)\cap H^{2}\left(\Omega \right)\right]\;\times \;\left[L^{2}\left(\Omega \right)\right]\;\times L^{2}\left(\Omega \right)$$

which endowed with the mapping 〈.;.〉 defined by:

(3.2) $$\begin{array}{c} <\left(u,v,\theta \right),\left(\tilde{u},\tilde{v},\tilde{\theta }\right)\geq \int_{\Omega }\left\{\rho^{-1}v\tilde{v}+\left(\lambda +2\mu \right)\nabla u.\nabla \tilde{u}+c^{-1}\theta \tilde{\theta }\right\}dx. \end{array}$$

Proposition 3.1 The mapping 〈;〉 defined on H by (3.2) is an inner product.

Proof. We show that 〈,〉 is bilinear and symmetric. Let $\left(u,v,\theta \right),\left(\tilde{u},\tilde{v},\tilde{\theta }\right),\left(u_{1},v_{1},\theta_{1}\right),\in H\times H\times H$ and α∈R.

$$<\alpha \left(u,v,\theta \right)+\left(u_{1},v_{1},\theta_{1}\right),\left(\tilde{u},\tilde{v},\tilde{\theta }\right)\geq \langle \left(\alpha u+u_{1},\alpha v+v_{1},\alpha \theta +\theta_{1}\right);\left(\tilde{u},\tilde{v},\tilde{\theta }\right)\rangle .$$

$$=\int_{\Omega }\left\{\rho^{-1}\left(\alpha v+v_{1}\right)\tilde{v}+\left(\lambda +2\mu \right)\left(\alpha \nabla u+\nabla u_{1}\right)\nabla \tilde{u}+c^{-1}\left(\alpha \theta +\theta_{1}\right)\tilde{\theta }\right\}dx.$$

$$=\int_{\Omega }\left\{\rho^{-1}\alpha v\tilde{v}+\rho^{-1}v_{1}\tilde{v}+\alpha \left(\lambda +2\mu \right)\nabla u\cdot \nabla \tilde{u}+\left(\lambda +2\mu \right)\nabla u_{1}\cdot \nabla \tilde{u}+\alpha c^{-1}\theta \tilde{\theta }+c^{-1}\theta_{1}\tilde{\theta }\right\}dx.$$

$$\begin{array}{cc} & =\int_{\Omega }\left\{\alpha \rho^{-1}v\tilde{v}+\alpha \left(\lambda +2\mu \right)\nabla u\cdot \nabla \tilde{u}+\alpha c^{-1}\theta \tilde{\theta }+\rho^{-1}v_{1}\tilde{v}+\left(\lambda +2\mu \right)\nabla u_{1}\cdot \nabla \tilde{u}+c^{-1}\theta_{1}\tilde{\theta }\right\}dx.\\ & =\alpha \int_{\Omega }\left\{\rho^{-1}v\tilde{v}+\left(\lambda +2\mu \right)\nabla u\cdot \nabla \tilde{u}+c^{-1}\theta \tilde{\theta }\right\}dx+\int_{\Omega }\left\{+\rho^{-1}v_{1}\tilde{v}+\left(\lambda +2\mu \right)\nabla u_{1}\cdot \nabla \tilde{u}+c^{-1}\theta_{1}\tilde{\theta }\right\}dx. \end{array}$$

$$=\alpha \langle \left(u,v,\theta \right),\left(\tilde{u},\tilde{v},\tilde{\theta }\right)\rangle +\langle \left(u_{1},v_{1},\theta_{1}\right),\left(\tilde{u},\tilde{v},\tilde{\theta }\right)\rangle .$$

$$<\left(u,v,\theta \right),\alpha \left(u_{1},v_{1},\theta_{1}\right)+\left(\tilde{u},\tilde{v},\tilde{\theta }\right)\geq \langle \left(u,v,\theta \right),\left(\alpha u_{1}+\tilde{u},\alpha v_{1}+\tilde{v},\alpha \theta_{1}+\tilde{\theta }\right)\rangle .$$

$$=\int_{\Omega }\left\{\rho^{-1}v\left(\alpha v_{1}+\tilde{v}\right)+\left(\lambda +2\mu \right)\nabla u\left(\alpha \nabla u_{1}+\nabla \tilde{u}\right)+c^{-1}\theta \left(\alpha \theta_{1}+\tilde{\theta }\right)\right\}dx.$$

$$=\int_{\Omega }\left\{\rho^{-1}v\tilde{v}+\alpha \rho^{-1}v_{1}\tilde{v}+\left(\lambda +2\mu \right)\nabla u\cdot \nabla \tilde{u}+\alpha \left(\lambda +2\mu \right)\nabla u_{1}\cdot \nabla \tilde{u}+\alpha c^{-1}\theta \tilde{\theta }+\alpha c^{-1}\theta_{1}\tilde{\theta }\right\}dx.$$

$$=\int_{\Omega }\left\{\rho^{-1}v\tilde{v}+\left(\lambda +2\mu \right)\nabla u\nabla \tilde{u}+c^{-1}\theta \tilde{\theta }+\alpha \rho^{-1}v_{1}\tilde{v}+\alpha \left(\lambda +2\mu \right)\nabla u_{1}\cdot \nabla \tilde{u}+\alpha c^{-1}\theta_{1}\tilde{\theta }\right\}dx.$$

$$=\int_{\Omega }\left\{\rho^{-1}v\tilde{v}+\left(\lambda +2\mu \right)\nabla u\nabla \tilde{u}+c^{-1}\theta \tilde{\theta }\right\}dx+\alpha \int_{\Omega }\left\{+\rho^{-1}v_{1}\tilde{v}+\left(\lambda +2\mu \right)\nabla u_{1}\cdot \nabla \tilde{u}+c^{-1}\theta_{1}\tilde{\theta }\right\}dx.$$

$$\leq \left(u,v,\theta \right),\left(\tilde{u},\tilde{v},\tilde{\theta }\right)>+\alpha \langle \left(u_{1},v_{1},\theta_{1}\right),\left(\tilde{u},\tilde{v},\tilde{\theta }\right)\rangle .$$

〈,〉 is bilinear.

$$<\left(u,v,\theta \right),\left(\tilde{u},\tilde{v},\tilde{\theta }\right)\geq \int_{\Omega }\left\{\rho^{-1}v\tilde{v}+\left(\lambda +2\mu \right)\nabla u.\nabla \tilde{u}+c^{-1}\theta \tilde{\theta }\right\}dx.$$

$$=\int_{\Omega }\left\{\rho^{-1}\tilde{v}v+\left(\lambda +2\mu \right)\nabla \tilde{u}.\nabla u+c^{-1}\tilde{\theta }\theta \right\}dx.$$

$$\leq \left(\tilde{u},\tilde{v},\tilde{\theta }\right),\left(u,v,\theta \right)>.$$

Then 〈,〉 is symmetric. We show that 〈,〉 is positive definite.

$$<\left(u,v,\theta \right),\left(u,v,\theta \right)\geq \int_{\Omega }\left\{\rho^{-1}vv+\left(\lambda +2\mu \right)\nabla u.\nabla u+c^{-1}\theta \theta \right\}dx.$$

$$=\int_{\Omega }\left\{\rho^{-1}{\left|v\right|}^{2}+\left(\lambda +2\mu \right){\left|\nabla u\right|}^{2}+c^{-1}{\left|\theta \right|}^{2}\right\}dx.$$

$\rho^{-1}>0$; (λ+2μ)>0 and $c^{-1}>0$ then

$(\rho^{-1}{\left|v\right|}^{2}+\left(\lambda +2\mu \right){\left|\nabla u\right|}^{2}+c^{-1}{\left|\theta \right|}^{2})\geq 0.$So

$$\int_{\Omega }\left\{\rho^{-1}{\left|v\right|}^{2}+\left(\lambda +2\mu \right){\left|\nabla u\right|}^{2}+c^{-1}{\left|\theta \right|}^{2}\right\}dx\geq 0.$$

We have 〈(u,v,θ),(u,v,θ)〉≥0. Moreover

$$<\left(u,v,\theta \right),\left(u,v,\theta \right)\geq 0\Leftrightarrow \int_{\Omega }\left\{\rho^{-1}{\left|v\right|}^{2}+\left(\lambda +2\mu \right){\left|\nabla u\right|}^{2}+c^{-1}{\left|\theta \right|}^{2}\right\}dx=0.$$

$$<\left(u,v,\theta \right),\left(u,v,\theta \right)\geq 0\Leftrightarrow \rho^{-1}{\left|v\right|}^{2}+\left(\lambda +2\mu \right){\left|\nabla u\right|}^{2}+c^{-1}{\left|\theta \right|}^{2}=0.$$

<(u,v,θ),(u,v,θ)≥0⇔v=∇u=θ=0.

∇u=0 then u is constant with respect to x. u(t,0)=0, then u=0. We have

<(u,v,θ),(u,v,θ)≥0⇔u=v=θ=0.

Conclusion: 〈;〉 is inner product on H.

Proposition 3.2 equipped with the inner product 〈;〉 defined by (3.2) is a Hilbert space.

Proof. H equipped with the inner product 〈;〉 defined by (3.2) is a prehilbertian space. We will show that H is complete. Let ${\left(u^{n},v^{n},\theta^{n}\right)}_{n\geq 0}$ be a sequence of elements of H.

$$\left(u^{n},v^{n},\theta^{n}\right)-{\left(u^{m},v^{m},\theta^{m}\right)}_{H}^{2}=\rho^{-1}v^{n}-{v^{m}}_{L^{2}\left(\Omega \right)}^{2}+\left(\lambda +2\mu \right)u^{n}-{u^{m}}_{H_{0}^{1}\left(\Omega \right)}^{2}+c^{-1}\theta^{n}-{\theta^{m}}_{L^{2}\left(\Omega \right)}^{2}.$$

Observe that therefore $\left(u^{n},v^{n},\theta^{n}\right)$ is a Cauchy sequence in H if and only if ${\left(u^{n}\right)}_{n}$ is a Cauchy sequence in $H^{2}\left(\Omega \right)\cap H_{0}^{1}\left(\Omega \right)$, ${\left(v^{n}\right)}_{n}$ is a Cauchy sequence in $L^{2}\left(\Omega \right)$ and ${\left(\theta^{n}\right)}_{n}$ is a Cauchy sequence in $L^{2}\left(\Omega \right)$. According to relation (3), the sequences $u^{n}$, $v^{n}$ and $\theta^{n}$ are from Cauchy in $H^{2}\left(\Omega \right)\cap H_{0}^{1}\left(\Omega \right)$, $L^{2}\left(\Omega \right)$ and $L^{2}\left(\Omega \right)$ respectively. $u^{n}$ is a Cauchy sequence in $H^{2}\left(\Omega \right)\cap H_{0}^{1}\left(\Omega \right)$ and since $H^{2}\left(\Omega \right)\cap H_{0}^{1}\left(\Omega \right)$ is complete, then there exists a rank $n_{1}$ from which $u^{n}$ converges to an element $l_{1}$ in $H^{2}\left(\Omega \right)\cap H_{0}^{1}\left(\Omega \right)$.

$v^{n}$ is a Cauchy sequence in $L^{2}\left(\Omega \right)$ and since $L^{2}\left(\Omega \right)$ is complete, then there exists a rank $n_{2}$ from which $v^{n}$ converges to an element $l_{2}$ in $L^{2}\left(\Omega \right)$.

$\theta^{n}$ is a Cauchy sequence in $L^{2}\left(\Omega \right)$ and since $L^{2}\left(\Omega \right)$ is complete, then there exists a rank $n_{3}$ from which $\theta^{n}$ converges to an element $l_{3}$ in $L^{2}\left(\Omega \right)$.

Taking $n^{\prime }=max\left(n_{1},n_{2},n_{3}\right)$, ${\left(u^{n},v^{n},\theta^{n}\right)}_{n}$ converges to the triplet $\left(l_{1},l_{2},l_{3}\right)$ belonging to H from rank $n^{\prime }$.

Conclusion: H is a Hilbert space.

Let us define the operator A:D(A)⊆H→H by

$$D\left(A\right)=\left[H^{2}\left(\Omega \right)\cap H_{0}^{1}\left(\Omega \right)\right]\;\times \;\left[H^{2}\left(\Omega \right)\cap H_{0}^{1}\left(\Omega \right)\right]\;\times \;\left[H^{2}\left(\Omega \right)\cap H_{0}^{1}\left(\Omega \right)\right],$$

and

$$A\left(\begin{array}{c} u\\ \begin{array}{c} v\\ \theta \end{array} \end{array}\right)=\;\left(\begin{array}{c} \rho^{-1}v_{\;}\\ \begin{array}{c} \left(\lambda +2\mu \right)\Delta u+m\nabla \theta \\ c^{-1}k\Delta \theta +c^{-1}\rho^{-1}\theta m\nabla v \end{array} \end{array}\right)$$

for each (u,v,θ)∈D(A). At this point, let us observe that the system (3.1) can be equivalently rewritten on the abstract form

(3.3) $$\begin{array}{c} \left\{ \begin{array}{cc} Z{}^{\prime }\left(t\right)=AZ\left(t\right) & t\geq 0\\ & Z\left(0\right)=\xi \end{array}\right. \end{array}$$

where $Z\left(t\right)\left(x\right)=\left(\begin{array}{c} u(t,x)\\ v(t,x)\\ \theta (t,x) \end{array}\right)$ and $\xi \left(x\right)=\left(\begin{array}{c} u_{0}\\ v_{0}\\ \theta_{0} \end{array}\right)$.

## Main results

### Existence and uniqueness of solution of problem (3.3)

Theorem 4.1 The operator -A is maximal monotone on H under the conditions $(\Theta m-ck^{\left(-1\right)})<0$ and $(\rho^{-1}m+\lambda +2\mu )>0$.

Proof. We will show that the operator I−A is surjective.

Let $(\tilde{u},\tilde{v},\tilde{\theta })\in H$ and (u,v,θ)∈D(A).

$$\left(I-A\right)\left(u,v,\theta \right)=\left(\tilde{u},\tilde{v},\tilde{\theta }\right)\Rightarrow$$

(4.1) $$\left\{ \begin{array}{c} u-\;\rho^{-1}v_{\;}=\;\tilde{u}\;\\ v-\;\left(\lambda +2\mu \right)\Delta u+m\nabla \Theta =\;\tilde{v}\;\\ \theta -\;c^{-1}k\Delta \theta +c^{-1}\rho^{-1}\Theta m\nabla v=\;\tilde{\theta \;}\; \end{array}\right.$$

Since

$$\theta -\;c^{-1}k\Delta \theta +c^{-1}\rho^{-1}\Theta m\nabla v=\;\tilde{\theta }$$

then

$$\Delta \theta -c\;k^{-1}\theta +k^{-1}\rho^{-1}\Theta m\nabla v=-ck^{\left(-1\right)}\tilde{\theta }.$$

$$\nabla v=\;\rho k\theta \;\Rightarrow \;\Delta \theta -c\;k^{-1}\Theta +\Theta m\theta =-c\;k^{-1}\;\tilde{\theta }$$

$$\Delta \theta +\left(\Theta m-c\;k^{-1}\right)\theta =g\left(t,x\right)$$

with $g\left(t,x\right)=-ck^{-1}\tilde{\theta }.$ The characteristic equation $r^{2}+\;\Theta m\;-c\;k^{-1}=0$ has two real solutions ω and –ω with ω $=\;\sqrt{-(\Theta m-c\;k^{-1})}$ and $(\Theta m-c\;k^{-1})<0$.

$$\theta \left(t,x\right)=C_{1}\left(t\right)e^{\omega x}+C_{2}\left(t\right)e^{-\omega x}+\int_{0}^{x}\left(C_{1}\left(t\right)e^{\omega \left(x-y\right)}+C_{2}\left(t\right)e^{-\omega \left(x-y\right)}\right)g\left(t,y\right)dy.$$

$$\theta \left(0,t\right)=0\Leftrightarrow C_{1}\left(t\right)+C_{2}\left(t\right)=0\Leftrightarrow C_{1}\left(t\right)=-C_{2}\left(t\right).$$

$$\theta \left(t,x\right)=C_{1}\left(t\right)\left(e^{\omega x}-e^{-\omega x}+C_{1}\left(t\right)\int_{0}^{x}(e^{\omega \left(x-y\right)}-e^{-\omega \left(x-y\right)}\right)g\left(t,y\right)dy.$$

$sh\left(\omega x\right)=\frac{e^{\omega x}-e^{-\omega x}}{2}$. Then

$$\theta \left(t,x\right)=2C_{1}\left(t\right)sh\left(\omega x\right)+2C_{1}\left(t\right)\int_{0}^{x}sh\left(\omega \left(x-y\right)\right)g\left(t,y\right)dy.$$

$u-\rho^{-1}v=\tilde{u}$ then

$$u-\rho^{-1}\left(\lambda +2\mu \right)\Delta u-\rho^{-1}m\nabla \theta =\tilde{u}.$$

$$\nabla u=\rho \theta \Rightarrow \theta =\rho^{-1}\nabla u.$$

$$\nabla u=\rho \theta \Rightarrow \nabla \theta =\;\rho^{-1}\Delta u.$$

Then $\nabla \theta =\rho^{-1}\Delta u.$

$$u-\rho^{-1}\left(\lambda +2\mu \right)\Delta u-\rho^{-1}m\nabla \theta =\tilde{u}.$$

$$u-\rho^{-1}\left(\lambda +2\mu \right)\Delta u-\rho^{-2}m\Delta u=\tilde{u}.$$

(4.2) $$u-\rho^{-1}\left(\rho^{-1}m+\lambda +2\mu \right)\Delta u=f\left(t,x\right),$$

with

$f\left(t,x\right)=\tilde{u}.$

We thus obtain a linear differential equation of the second order with second member. The homogeneous equation has for characteristic equation

$-\rho^{-1}\left(\rho^{-1}m+\lambda +2\mu \right)r^{2}+1=0.$

Since $(\rho^{-1}m+\lambda +2\mu )>0$, then the characteristic equation admits two real solutions $\omega_{1}$ and $-\omega_{1}$ with

$\omega_{1}=\sqrt{\rho {\left(\rho^{-1}m+\lambda +2\mu \right)}^{-1}}$. Consequently the differential equation (4.2) has a unique solution u gived by:

$$u\left(t,x\right)=C_{1}\left(t\right)e^{\omega_{1}x}+C_{2}\left(t\right)e^{-\omega_{1}x}+\int_{0}^{x}\left(C_{1}\left(t\right)e^{\omega_{1}\left(x-y\right)}+C_{2}\left(t\right)e^{-\omega_{1}\left(x-y\right)}\right)f\left(t,y\right)dy.$$

Where $C_{1}$ and $C_{2}$ are real constants. $u\left(0,t\right)=0\Leftrightarrow C{\left(t\right)}_{1}+C_{2}\left(t\right)=0\Leftrightarrow C_{1}\left(t\right)=-C_{2}\left(t\right)$.

$$u\left(t,x\right)=C_{1}\left(t\right)\left(e^{\omega_{1}x}-e^{-\omega_{1}x}+C_{1}\left(t\right)\int_{0}^{x}(e^{\omega_{1}\left(x-y\right)}-e^{-\omega_{1}\left(x-y\right)}\right)f\left(t,y\right)dy.$$

$$u\left(t,x\right)=2C_{1}\left(t\right)sh\left(\omega_{1}x\right)+2C_{1}\left(t\right)\int_{0}^{x}sh\left(\omega_{1}\left(x-y\right)\right)f\left(t,y\right)dy.$$

So, for any triplet $(\tilde{u},\tilde{v},\tilde{\theta })\in H$, there exists a triplet (u,v,θ)∈D(A) such that

$$\left(I-A\right)\left(u,v,\theta \right)=\left(\tilde{u},\tilde{v},\tilde{\theta }\right)$$

that is to say I−A is surjective and Im(I−A)=H.

(4.3) $$\begin{array}{c} <\left(u,v,\theta \right),\left(\tilde{u},\tilde{v},\tilde{\theta }\right)\geq \int_{\Omega }\left\{\rho^{-1}v\tilde{v}+\left(\lambda +2\mu \right)\nabla u\cdot \nabla \tilde{u}+c^{-1}\theta \tilde{\theta }\right\}dx. \end{array}$$

$$A\left(\begin{array}{c} u\\ \begin{array}{c} v\\ \theta \end{array} \end{array}\right)=\;\left(\begin{array}{c} \rho^{-1}v\\ \begin{array}{c} \left(\lambda +2\mu \right)\Delta u+m\nabla \theta \\ c^{-1}k\Delta \theta +c^{-1}\rho^{-1}\Theta m\nabla v \end{array} \end{array}\right)$$

$$<A\left(u,v,\theta \right),\left(u,v,\theta \right)\geq \int_{\Omega }\left\{\rho^{-1}\left[\left(\lambda +2\mu \right)\Delta u+m\nabla \theta \right]v+\rho^{-1}\left[\left(\lambda +2\mu \right)\nabla u\cdot \nabla v\right]\right\}+$$

$$c^{-2}k\theta .\Delta \theta +c^{-2}\rho^{-1}\Theta m\theta .\nabla vdx$$

$$\begin{array}{c} <A\left(u,v,\theta \right),\left(u,v,\theta \right)\geq \rho^{-1}\int_{\Omega }\left(\lambda +2\mu \right)v\cdot \Delta udx+\rho^{-1}m\int_{\Omega }v\cdot \nabla \theta dx+ \end{array}$$

(4.4) $$\rho^{-1}\int_{\Omega }\left(\lambda +2\mu \right)\nabla u\cdot \nabla vdx+\;c^{-2}k\int_{\Omega }\theta \cdot \Delta \theta dx\;+\;c^{-2}\rho^{-1}\Theta m\int_{\Omega }\theta \cdot \nabla vdx$$

(4.5) $$\begin{array}{c} \rho^{-1}\int_{\Omega }\left(\lambda +2\mu \right)v\cdot \Delta udx=\rho^{-1}{\left[\left(\lambda +2\mu \right)v\cdot \nabla u\right]}_{\partial \Omega }-\rho^{-1}\int_{\Omega }\left(\lambda +2\mu \right)\nabla u\cdot \nabla vdx. \end{array}$$

(4.6) $$\begin{array}{c} \rho^{-1}\int_{\Omega }\left(\lambda +2\mu \right)v\cdot \Delta udx=-\rho^{-1}\int_{\Omega }\left(\lambda +2\mu \right)\nabla u\cdot \nabla vdx. \end{array}$$

(4.7) $$\begin{array}{c} \rho^{-1}m\int_{\Omega }v\cdot \nabla \theta dx=\rho^{-1}m{\left[\theta \cdot v\right]}_{\partial \Omega }-\rho^{-1}m\int_{\Omega }\theta \cdot \nabla \theta vdx. \end{array}$$

(4.8) $$\begin{array}{c} \rho^{-1}m\int_{\Omega }v\cdot \nabla \theta dx=-\rho^{-1}m\int_{\Omega }\theta \cdot \nabla vdx. \end{array}$$

(4.9) $$\begin{array}{c} c^{-2}k\int_{\Omega }\theta \cdot \Delta \theta dx=c^{-2}k{\left[\theta \cdot \nabla \theta \right]}_{\partial \Omega }-c^{-2}k\int_{\Omega }\nabla \theta \cdot \nabla \theta dx. \end{array}$$

(4.10) $$\begin{array}{c} c^{-2}k\int_{\Omega }\theta \cdot \Delta \theta dx=-c^{-2}k\int_{\Omega }\nabla \theta \cdot \nabla \theta dx. \end{array}$$

$$\begin{array}{c} <A\left(u,v,\Omega \right),\left(u,v,\theta \right)\geq -\rho^{-1}\left(\lambda +2\mu \right)\int_{\Omega }\nabla u\cdot \nabla vdx-\rho^{-1}m\int_{\Omega }\theta \cdot \nabla vdx+ \end{array}$$

(4.11) $$\rho^{-1}\left(\lambda +2\mu \right)\int_{\Omega }\nabla u\cdot \nabla vdx-c^{-2}k\int_{\Omega }\nabla \theta \cdot \nabla \theta dx+\;-c^{-2}\rho^{-1}\Theta m\int_{\Omega }\theta \cdot \nabla vdx.$$

$$\frac{\Theta }{c^{2}}=1\;\Rightarrow \;c^{-2}\rho^{-1}\Theta m=\;m\rho^{-1}$$

$$\begin{array}{c} <A\left(u,v,\theta \right),\left(u,v,\theta \right)\geq -\rho^{-1}\left(\lambda +2\mu \right)\int_{\Omega }\nabla u\cdot \nabla vdx-\rho^{-1}m\int_{\Omega }\theta \cdot \nabla vdx+ \end{array}$$

(4.12) $$\rho^{-1}\left(\lambda +2\mu \right)\int_{\Omega }\nabla u\cdot \nabla vdx-c^{-2}k\int_{\Omega }\nabla \theta \cdot \nabla \theta dx+\rho^{-1}m\int_{\Omega }\theta \cdot \nabla vdx.$$

then

(4.13) $$\begin{array}{c} <A\left(u,v,\theta \right),\left(u,v,\theta \right)\geq -c^{-2}k\int_{\Omega }\nabla \theta \cdot \nabla \theta dx. \end{array}$$

(4.14) $$\begin{array}{c} <A\left(u,v,\theta \right),\left(u,v,\theta \right)>=-c^{-2}k\int_{\Omega }\nabla \theta^{2}dx. \end{array}$$

Since k$\;c^{-2}\;$> 0, then

〈A(u,v,θ),(u,v,θ)〉≤0.So

<−A(u,v,θ),(u,v,θ)>=−〈A(u,v,θ),(u,v,θ)〉≥0.

Consequently, the operator −A is monotone.

The operator −A is monotone and Im(I−A)=H then the operator −A is maximal monotone.

Conclusion: Using the Theorem, the problem (3.3) admits a unique solution

Z(t)(x)=T(t)ξ(x) for all ξ∈D(A) where ${\left(T(t)\right)}_{t\geq 0}$ is the $C_{0}$-semi group of contractions generated by A.

#### Stability of the solution of the initial value problem

We will consider the case where m=0 and the case where the displacement u and the momentum v are proportional to within one constant ie u = $\rho^{-1}v$.

For m=0, we have the following system

(4.15) $$\begin{array}{c} \left\{ \begin{array}{cc} u_{t}=\rho^{-1}v_{t} & \left(t,x\right)\in R_{+}\times \Omega \\ v_{t}=\left(\lambda +2\mu \right)\Delta u & \left(t,x\right)\in R_{+}\times \Omega \\ \theta_{t}=c^{-1}k\Delta \theta & \left(t,x\right)\in R_{+}\times \Omega \\ u\left(t,x\right)=\theta \left(t,x\right)=0 & \left(t,x\right)\in R_{+}\times \partial \Omega \\ u\left(0,x\right)=u_{0}\left(x\right) & x\in \Omega \\ v_{0}\left(x\right)=v\left(0,x\right) & x\in \Omega \\ \theta \left(0,x\right)=\theta_{0}\left(x\right) & x\in \Omega \end{array}\right. \end{array}$$

Proposition 4.2 $$\int_{0}^{\infty }T\left(t\right)\xi^{2}dt<\infty$$

for every ξ∈H.

Let ${\left(\lambda_{n}\right)}_{n\geq 1}$ be an increasing sequence of eigenvalues of the operator Δ. The eigenvectors ${\left(e_{n}\right)}_{n}$ associated with

$\lambda_{n}\left(-\Delta e_{n}=\lambda_{n}e_{n}\right)$ form a Hilbertian basis. Let ${\left(T_{1}\left(t\right)\right)}_{t\geq 0}$ be the $C_{0}$-semi-group generated by the operator Δ with $D\left(\Delta \right)=H^{2}\left(\Omega \right)\cap H_{0}^{1}\left(\Omega \right)$ and Ω=[0,π]. We pose $f=\sum_{n=1}^{\infty }\left(f,e_{n}\right)e_{n}$.

We have

$T_{1}\left(t\right)f=\sum_{n=1}^{\infty }\left(f,e_{n}\right)e_{n}e^{-n^{2}t}.$

Lemma 4.3 Let ${\left(T^{\prime }\left(t\right)\right)}_{t\geq 0}$ be the $C_{0}$-semi-group generated by the operator A. Then for all α>0, αA generates ${\left(T^{\prime }\left(\alpha t\right)\right)}_{t\geq 0}$.

Since the constants $c^{-1}k$ and $\rho^{-1}\left(\lambda +2\mu \right)$ are strictly positive, then $c^{-1}k\Delta$ and $\rho^{-1}\left(\lambda +2\mu \right)\Delta$ generate ${\left(T_{1}\left(c^{-1}kt\right)\right)}_{t\geq 0}$ and ${\left(T_{1}\left(\rho^{-1}\left(\lambda +2\mu \right)t\right)\right)}_{t\geq 0}$ respectively.

Since $T_{1}\left(t\right)f=\sum_{n=1}^{\infty }\left(f,e_{n}\right)e_{n}e^{-n^{2}t}$, then

$$T_{1}\left(c^{-1}kt\right)e_{n}=\sum_{n=1}^{\infty }e^{-c^{-1}kn^{2}t}e_{n}$$

and

$$T_{1}\left(\rho^{-1}\left(\lambda +2\mu \right)t\right)=\sum_{n=1}^{\infty }e^{-\rho^{-1}\left(\lambda +2\mu \right)n^{2}t}e_{n}.$$

The equation $\theta_{t}=c^{-1}k\Delta \theta$ with θ(0,t)=θ(π,t)=0 and $\theta \left(x,0\right)=\theta_{0}\left(x\right)$ admits a unique solution defined by

$$\theta \left(t,x\right)=T_{1}\left(c^{-1}kt\right)\theta_{0}\left(x\right).$$

The equation $u_{t}=\rho^{-1}\left(\lambda +2\mu \right)\Delta u$ with u(0,t)=u(π,t)=0 and $u\left(x,0\right)=u_{0}\left(x\right)$ admits a unique solution defined by

$$u\left(t,x\right)=T_{1}\left(\rho^{-1}\left(\lambda +2\mu \right)t\right)u_{0}\left(x\right).$$

$$v_{t}\left(t,x\right)=\rho u_{t}\left(t,x\right)\Rightarrow v\left(t,x\right)=\rho u\left(t,x\right)+c.$$

v(t,0)=u(t,0)=0⇒c=0.

Then

$$v\left(t,x\right)=\rho u\left(t,x\right)=T_{1}\left(\rho^{-1}\left(\lambda +2\mu \right)t\right)\rho u_{0}\left(x\right)=T_{1}\left(\rho^{-1}\left(\lambda +2\mu \right)t\right)v_{0}\left(x\right)$$

with $v_{0}\left(x\right)=\rho u_{0}\left(x\right).$We have:

T(t)ξ(x)=(u(t,x);v(t,x),θ(t,x)).

$$T\left(t\right)\xi {\left(x\right)}_{H}^{2}\leq \left(u(t,x);v(t,x);\theta (t,x)\right);\left(u(t,x);v(t,x);\theta (t,x)\right)>.$$

$$\int_{\Omega }\left\{\rho^{-1}{\left|v(t,x)\right|}^{2}+\left(\lambda +2\mu \right){\left|\nabla u(t,x)\right|}^{2}+c^{-1}{\left|\theta (t,x)\right|}^{2}\right\}dx.$$

$$=\rho^{-1}\int_{\Omega }{\left|v(t,x)\right|}^{2}dx+\left(\lambda +2\mu \right)\int_{\Omega }{\left|\nabla u(t,x)\right|}^{2}dx+c^{-1}\int_{\Omega }{\left|\theta (t,x)\right|}^{2}dx.$$

$$=\rho^{-1}\int_{\Omega }{|T_{1}\left(\rho^{-1}\left(\lambda +2\mu \right)t\right)v_{0}\left(x\right)|}^{2}dx+\left(\lambda +2\mu \right)\int_{\Omega }{|T_{1}\left(\rho^{-1}\left(\lambda +2\mu \right)t\right)\nabla u_{0}\left(x\right)|}^{2}dx+c^{-1}\int_{\Omega }{|T_{1}\left(c^{-1}kt\right)\theta_{0}\left(x\right)|}^{2}dx.$$

$$\leq \rho^{-1}{|T_{1}\left(\rho^{-1}\left(\lambda +2\mu \right)t\right)|}^{2}\int_{\Omega }{|v_{0}\left(x\right)|}^{2}dx+\left(\lambda +2\mu \right){|T_{1}\left(\rho^{-1}\left(\lambda +2\mu \right)t\right)|}^{2}\int_{\Omega }{|\nabla u_{0}\left(x\right)|}^{2}dx+c^{-1}{|T_{1}\left(c^{-1}kt\right)|}^{2}\int_{\Omega }{|\theta_{0}\left(x\right)|}^{2}dx.$$

$$\leq \rho^{-1}\sum_{n=1}^{\infty }e^{-2\rho^{-1}\left(\lambda +2\mu \right)n^{2}t}v_{0}{\left(x\right)}_{L^{2}\left(\Omega \right)}^{2}+\left(\lambda +2\mu \right)\sum_{n=1}^{\infty }e^{-2\rho^{-1}\left(\lambda +2\mu \right)n^{2}t}u_{0}{\left(x\right)}_{H_{0}^{1}\left(\Omega \right)}^{2}+c^{-1}\sum_{n=1}^{\infty }e^{-2c^{-1}kn^{2}t}\theta_{0}{\left(x\right)}_{L^{2}\left(\Omega \right)}^{2}.$$

We have $H=H^{2}\left(\Omega \right)\cap H_{0}^{1}\left(\Omega \right)\;\times L^{2}\left(\Omega \right)\;\times L^{2}\left(\Omega \right)$ so for all ξ∈H, $u_{0}\left(x\right)\in H^{2}\left(\Omega \right)\cap H_{0}^{1}\left(\Omega \right),$ $v_{0}\left(x\right)\in L^{2}\left(\Omega \right),$ and $\theta_{0}\left(x\right)\in L^{2}\left(\Omega \right)$. Since Ω a bounded open, then there exists α>0, β>0, γ>0 such that:

$v_{0}{\left(x\right)}_{L^{2}\left(\Omega \right)}^{2}<\alpha$, $u_{0}{\left(x\right)}_{H_{0}^{1}\left(\Omega \right)}^{2}<\beta$ and $\theta_{0}{\left(x\right)}_{L^{2}\left(\Omega \right)}^{2}<\gamma$.

$$\rho^{-1}\sum_{n=1}^{\infty }e^{-2\rho^{-1}\left(\lambda +2\mu \right)n^{2}t}v_{0}{\left(x\right)}_{L^{2}\left(\Omega \right)}^{2}<\alpha \rho^{-1}\sum_{n=1}^{\infty }e^{-2\rho^{-1}\left(\lambda +2\mu \right)n^{2}t}.$$

$$\left(\lambda +2\mu \right)\sum_{n=1}^{\infty }e^{-2\rho^{-1}\left(\lambda +2\mu \right)n^{2}t}u_{0}{\left(x\right)}_{H_{0}^{1}\left(\Omega \right)}^{2}<\beta \left(\lambda +2\mu \right)\sum_{n=1}^{\infty }e^{-2\rho^{-1}\left(\lambda +2\mu \right)n^{2}t}.$$

$$c^{-1}\sum_{n=1}^{\infty }e^{-2c^{-1}kn^{2}t}\theta_{0}{\left(x\right)}_{L^{2}\left(\Omega \right)}^{2}<\gamma c^{-1}\sum_{n=1}^{\infty }e^{-2c^{-1}kn^{2}t}.$$

We have:

$$T\left(t\right)\xi_{H}^{2}\leq \alpha \rho^{-1}\sum_{n=1}^{\infty }e^{-2\rho^{-1}\left(\lambda +2\mu \right)n^{2}t}+\beta \left(\lambda +2\mu \right)\sum_{n=1}^{\infty }e^{-2\rho^{-1}\left(\lambda +2\mu \right)n^{2}t}+\gamma c^{-1}\sum_{n=1}^{\infty }e^{-2c^{-1}kn^{2}t}.$$

$$\int_{0}^{\infty }T\left(t\right)\xi_{H}^{2}dt=\underset{\epsilon \to +\infty }{\text{lim}}\int_{0}^{\epsilon }T\left(t\right)\xi_{H}^{2}dt.$$

$$\underset{\epsilon \to +\infty }{\text{lim}}\left({\left[\frac{-\alpha \rho^{-1}}{2\rho^{-1}\left(\lambda +2\mu \right)n^{2}}e^{-2\rho^{-1}\left(\lambda +2\mu \right)n^{2}t}\right]}_{0}^{\epsilon }\right)=\underset{\epsilon \to +\infty }{\text{lim}}\left(\frac{-\alpha \rho^{-1}}{2\rho^{-1}\left(\lambda +2\mu \right)n^{2}}e^{-2\rho^{-1}\left(\lambda +2\mu \right)n^{2}\epsilon }+\frac{\alpha }{2\left(\lambda +2\mu \right)n^{2}}\right).$$

$$\underset{\epsilon \to +\infty }{\text{lim}}\left({\left[\frac{-\alpha \rho^{-1}}{2\rho^{-1}\left(\lambda +2\mu \right)n^{2}}e^{-2\rho^{-1}\left(\lambda +2\mu \right)n^{2}t}\right]}_{0}^{\epsilon }\right)=\frac{\alpha }{2\left(\lambda +2\mu \right)n^{2}}.$$

$$\underset{\epsilon \to +\infty }{\text{lim}}\left({\left[\frac{-\beta \left(\lambda +2\mu \right)}{2\rho^{-1}\left(\lambda +2\mu \right)n^{2}}e^{-2\rho^{-1}\left(\lambda +2\mu \right)n^{2}t}\right]}_{0}^{\epsilon }\right)=\underset{\epsilon \to +\infty }{\text{lim}}\left(-\frac{\beta \left(\lambda +2\mu \right)}{2\rho^{-1}\left(\lambda +2\mu \right)n^{2}}e^{-2\rho^{-1}\left(\lambda +2\mu \right)n^{2}\epsilon }+\frac{\beta \rho }{2n^{2}}\right).$$

$$=\frac{\beta \rho }{2n^{2}}.$$

$$\underset{\epsilon \to +\infty }{\text{lim}}\left({\left[\frac{-\gamma c^{-1}}{2c^{-1}kn^{2}}e^{-2c^{-1}kn^{2}t}\right]}_{0}^{\epsilon }\right)=\underset{\epsilon \to +\infty }{\text{lim}}\left(-\frac{\gamma c^{-1}}{2c^{-1}kn^{2}}e^{-2c^{-1}kn^{2}\epsilon }+\frac{\gamma }{2kn^{2}}\right).$$

$$=\frac{\gamma }{2kn^{2}}.$$

$$\int_{0}^{\infty }T\left(t\right)\xi_{H}^{2}dt\leq \sum_{n=1}^{\infty }\left(\frac{\alpha }{2\left(\lambda +2\mu \right)n^{2}}+\frac{\beta \rho }{2n^{2}}+\frac{\gamma }{2kn^{2}}\right).$$

$$\int_{0}^{\infty }T\left(t\right)\xi_{H}^{2}dt<\infty .$$

for all ξ∈H. Conclusion: ${\left(T(t)\right)}_{t\geq 0}$ is uniformly exponentially stable according the proposition 2.7

## Declaration of competing interest

The authors declare that they have no known competing financial interests or personal relationships that could have appeared to influence the work reported in this paper.
